# The molecular biology of peritoneal metastatic disease

**DOI:** 10.15252/emmm.202215914

**Published:** 2023-01-26

**Authors:** Sanne Bootsma, Maarten F Bijlsma, Louis Vermeulen

**Affiliations:** ^1^ Laboratory for Experimental Oncology and Radiobiology, Center for Experimental and Molecular Medicine Amsterdam UMC, Location University of Amsterdam Amsterdam The Netherlands; ^2^ Cancer Center Amsterdam, Cancer Biology Amsterdam The Netherlands; ^3^ Amsterdam Gastroenterology Endocrinology Metabolism Amsterdam The Netherlands; ^4^ Oncode Institute Amsterdam The Netherlands

**Keywords:** gastrointestinal cancer, metastasis, peritoneum, tumor biology, tumor microenvironment, Cancer, Molecular Biology of Disease

## Abstract

Peritoneal metastases are a common form of tumor cell dissemination in gastrointestinal malignancies. Peritoneal metastatic disease (PMD) is associated with severe morbidity and resistance to currently employed therapies. Given the distinct route of dissemination compared with distant organ metastases, and the unique microenvironment of the peritoneal cavity, specific tumor cell characteristics are needed for the development of PMD. In this review, we provide an overview of the known histopathological, genomic, and transcriptomic features of PMD. We find that cancers representing the mesenchymal subtype are strongly associated with PMD in various malignancies. Furthermore, we discuss the peritoneal niche in which the metastatic cancer cells reside, including the critical role of the peritoneal immune system. Altogether, we show that PMD should be regarded as a distinct disease entity, that requires tailored treatment strategies.

## Graphical abstract

In this review, S. Bootsma, M. Bijlsma, and L. Vermeulen provide an overview of the histopathological, genomic, and transcriptomic features of peritoneal metastases and argue that peritoneal metastatic disease should be considered a distinct disease entity.

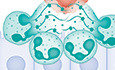

**Glossary**
Consensus Molecular Subtypes (CMS): Gene expression based classification system in colorectal cancer
Cytoreductive Surgery and Hyperthermic Intraperitoneal Chemotherapy (CRS‐HIPEC): Combination treatment of surgery and chemotherapy. During the operation, all visible tumors are removed, followed by flushing of the abdominal cavity with heated chemotherapy
Epithelial‐mesenchymal transition (EMT): Process in which epithelial cells acquire mesenchymal characteristics, which is associated with increased invasive behavior in cancer cells
M1/M2 macrophages: Two types of specialized immune cells. M1 macrophages have antitumor effects, while M2 macrophages promote tumor growth
Mesothelial cells: Layer of cells on the surface of the peritoneum that lines the intra‐abdominal organs. Cancer cells attach to mesothelial cells in the process of forming PM
Omentum: Fold of peritoneum and fatty tissue that hangs down over the stomach and the intestines
Synchronous/metachronous metastases: Metastases are discovered at the same time (synchronous) or at least 3 months after (metachronous) the diagnosis of the primary tumor

## Introduction

**Figure 1:**
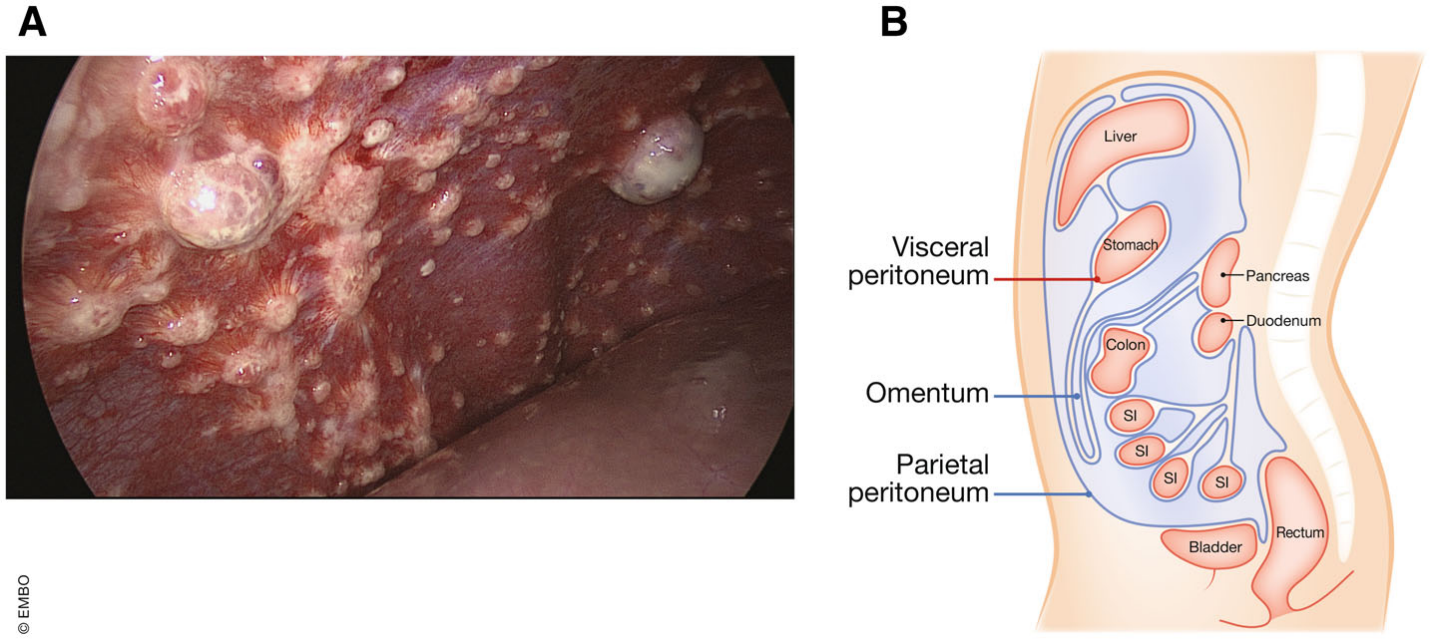
Anatomy of peritoneal metastases (A) Diaphragmatic peritoneal metastases originating from colorectal cancer imaged during diagnostic laparoscopy. Scale approximately 1:2. Image courtesy of I. de Hingh. (B) Sagittal view of the abdominal cavity. The abdominopelvic wall and diaphragm are lined by the parietal peritoneum, whereas the visceral peritoneum lines the organs. The omentum is the largest peritoneal fold. SI, small intestine.

Peritoneal metastases (PM) occur predominantly, but not exclusively, in abdominal malignancies such as colorectal, gastric, ovarian, and pancreatic cancer (Fig 1A). Patients with peritoneal metastatic disease (PMD) typically suffer from abdominal pain, weight loss, and an accumulation of fluid in the abdominal cavity referred to as ascites. The occurrence of PMD is associated with a poorer outcome as compared to metastatic disease involving other organs, such as the liver or the lungs (Franko *et al*, 2016). Peritoneal metastatic disease is typically diagnosed in an advanced stage of disease. However, in selected patients with limited disease burden, cytoreductive surgery with hyperthermic intraperitoneal chemotherapy (CRS‐HIPEC) can be performed, yet recurrence rates are high and so is the associated morbidity of this procedure (Verwaal *et al*, 2004; Breuer *et al*, 2021). To understand why PM respond poorly to current treatments, it is key to unravel their molecular biology and the interaction with the local microenvironment. This will ultimately provide leads for the improvement of currently employed therapies and aid the development of novel targeted strategies. In this review, we will discuss the molecular features that enable cells to undergo and survive the peritoneal metastatic cascade. We will focus on PM derived from three of the most common primary gastrointestinal malignancies: colorectal cancer (CRC), gastric cancer (GC), and pancreatic ductal adenocarcinoma (PDAC). By highlighting the commonalities of PM across different cancer types, we demonstrate that PMD should be regarded as a distinct disease entity.

### Incidence and prognosis

In the last decade, improved population‐based registration databases and cohort studies have given insight into the incidence of PM and the prognosis of patients suffering from this condition, of which an overview is given in Table 1. As becomes clear, PM occur frequently and are especially in the synchronous setting associated with a poor prognosis. In all described tumor types, an increase in patients with PM has been reported over the years (Koemans *et al*, 2021; Lurvink *et al*, 2021; Rijken *et al*, 2021). This can be attributed to better diagnostic techniques, increased awareness, improved registration, and longer survival after primary tumor diagnosis. The presented incidences are most likely still underreported, especially in the metachronous situation as diagnosing PM by imaging techniques is challenging and reported locations of metastases may be inaccurate in case of widespread disease.

**Table 1:** Incidence and survival of patients with PM.

	Incidence (% PM+ of all patients with CRC/GC/PDAC)	Overall survival PM patients (months)	Reference
CRC			
Synchronous	4.3–7.7%	7–8.1	Jayne *et al* (2002), Segelman *et al* (2012), van der Geest *et al* (2015) and Bakkers *et al* (2021)
Metachronous	4.2–4.8%	12–28	
GC			
Synchronous	10–21%	2.1–9.4	Riihimäki *et al* (2016), Choi *et al* (2020) and Koemans *et al* (2021)
Metachronous	Not reported	Not reported	
PDAC			
Synchronous	14%	1.9	Rijken *et al* (2021)
Metachronous	13.5%	14.1	Tanaka *et al* (2019)

CRC, colorectal cancer; GC, gastric cancer; PDAC, pancreatic ductal adenocarcinoma; PM, peritoneal metastases.

### Anatomy

The peritoneum is a serous membrane lining the abdominal cavity. It is composed of an outer layer of connective tissue and an inner layer of mesothelial cells, to which tumor cells adhere in the process of forming PM. The peritoneum can be divided into two parts: the parietal peritoneum, lining the abdominopelvic wall and the diaphragm, and the visceral peritoneum, lining the intra‐abdominal organs (Fig 1B). There are several peritoneal folds, of which the double‐folded greater omentum is the most apparent. This apron‐like, adipose‐rich tissue is attached to the stomach and covers a large portion of the abdominal cavity. The omentum plays an important role in the peritoneal immune system and is a common site for PM, which might be related to its rich vascularization and abundance of adipocytes, which can provide fatty acids to fuel tumor cell growth (Nieman *et al*, 2011). Activated neutrophils in the omentum form a premetastatic niche by extruding chromatin webs called neutrophil extracellular traps (NETs) (Lee *et al*, 2019). Furthermore, the omentum contains secondary lymphoid organs with dense capillary networks known as milky spots (Gerber *et al*, 2006; Rangel‐Moreno *et al*, 2009). The mesothelial cells in these spots have high levels of adhesion molecules, which promotes tumor cell attachment (Cui *et al*, 2002).

The peritoneal cavity is defined as the virtual space between the parietal and visceral peritoneum. In the physiological situation, it contains approximately 50–100 ml of peritoneal fluid. This liquid helps the organs, in particular the bowel, to move without friction. It contains water, electrolytes, immune cells, and humoral components such as complement C3 and C4 to resist infection (Isaza‐Restrepo *et al*, 2018). Lymphatic stomata, small fenestrations in the mesothelial surface that are in direct contact with lymphatic vessels, allow drainage of peritoneal fluid (Wassilev *et al*, 1998). In case of PMD, malignant ascites formation can occur due to impaired drainage and altered vascular permeability associated with a local inflammatory‐like response (Ayantunde & Parsons, 2007; Sangisetty & Miner, 2012). Diaphragmatic movement and gravity ensure a constant flow of the peritoneal fluid, which also influences the pattern of cancer cell spread once they have detached from the primary tumor (Pannu & Oliphant, 2015).

## Peritoneal metastatic cascade

### Dissemination

**Figure 2:**
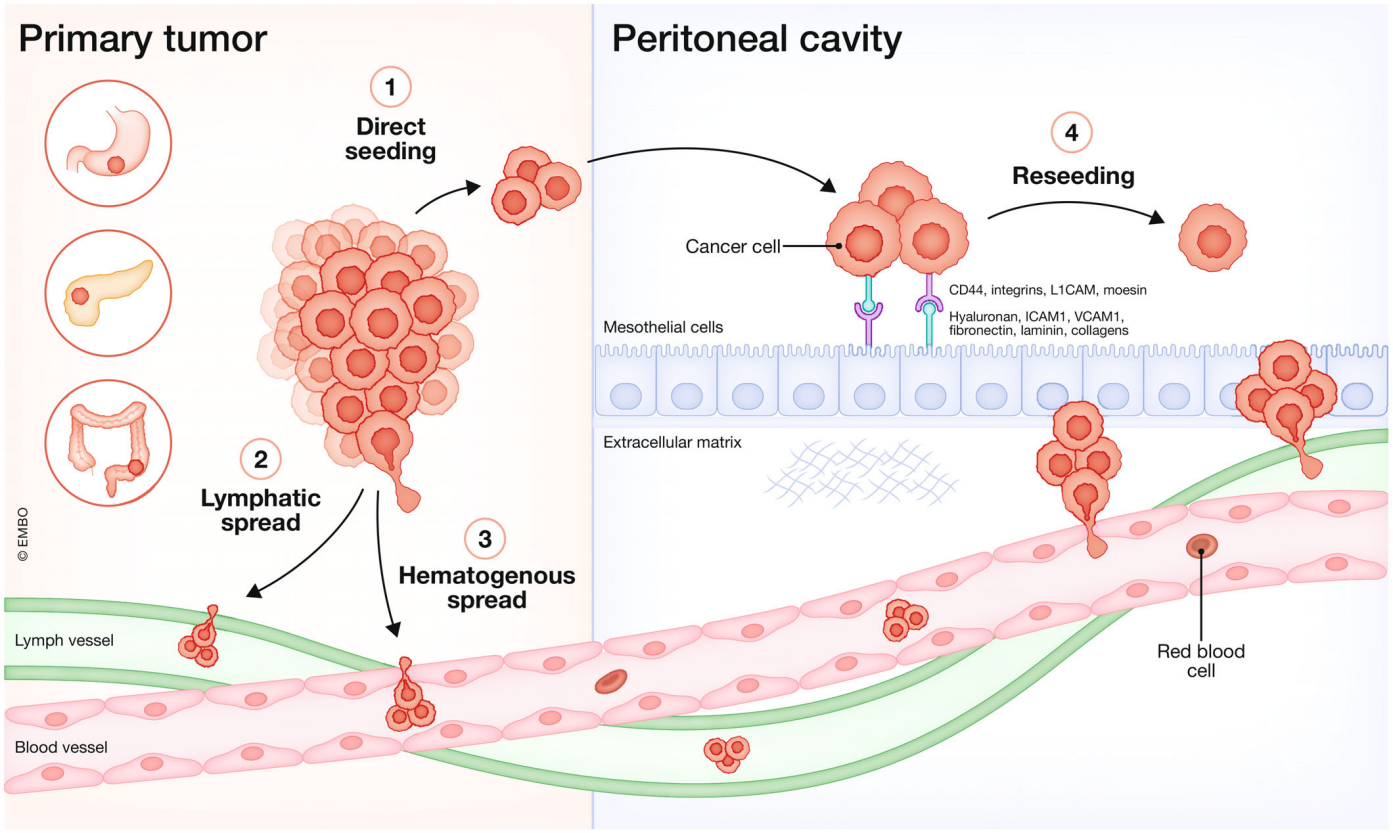
Routes of peritoneal metastases (PM) formation Cancer cell spread from the primary tumor to the peritoneum can occur via different routes: (1) direct seeding from the primary tumor into the peritoneal cavity, (2) via the lymphatic system, (3) via the hematogenous system, and (4) reseeding from existing PM lesions. Cancer cells interact with mesothelial cells via adhesion molecules. Created with BioRender.com.

The best described route of PM formation is direct invasion of cancer cells from the primary tumor into the peritoneal cavity. This can happen as a result of tumor‐cell intrinsic and microenvironmental factors that lead to detachment from the primary tumor and penetration through the serosa (Tanaka *et al*, 2017), or because of tissue damage and tumor cell spillage during surgery. Spread can occur directly to nearby structures such as the omentum in case of a primary tumor in the colon, or via the peritoneal fluid or ascites. Furthermore, tumor cells from already established peritoneal lesions can detach, spread, and reattach at a different location on the peritoneum (McPherson *et al*, 2016). The occurrence of PM from extraperitoneal organs such as breast cancer or melanoma suggests that tumor cells also reach the peritoneum via the lymphatic or hematogenous system (Flanagan *et al*, 2018). Tumor cells already present within the peritoneal cavity redistribute via these routes as well. Extensive regional lymph node involvement is considered a risk factor for the development of PM in nonserosa invasive GC (Huang *et al*, 2013) and CRC (Bhatt *et al*, 2019). As for the hematogenous route, a parabiosis mouse model of ovarian cancer, where mice share the same circulation, demonstrated the formation of omental metastases via the bloodstream (Pradeep *et al*, 2014). Furthermore, intracardiac injection of ovarian and CRC tumor cells led to omental tumor formation. This was regulated by the interaction between receptor tyrosine‐protein kinase erbB‐3 (HER3) on the tumor cells, and elevated levels of its ligand Neuregulin‐1 in the omentum (Pradeep *et al*, 2014). Also in patients, metastatic lesions of CRC and GC were found along, and even in the blood vessels of the peritoneum (Ge *et al*, 2017). Thus, different dissemination routes can lead to PM formation (Fig 2).

**Figure 3:**
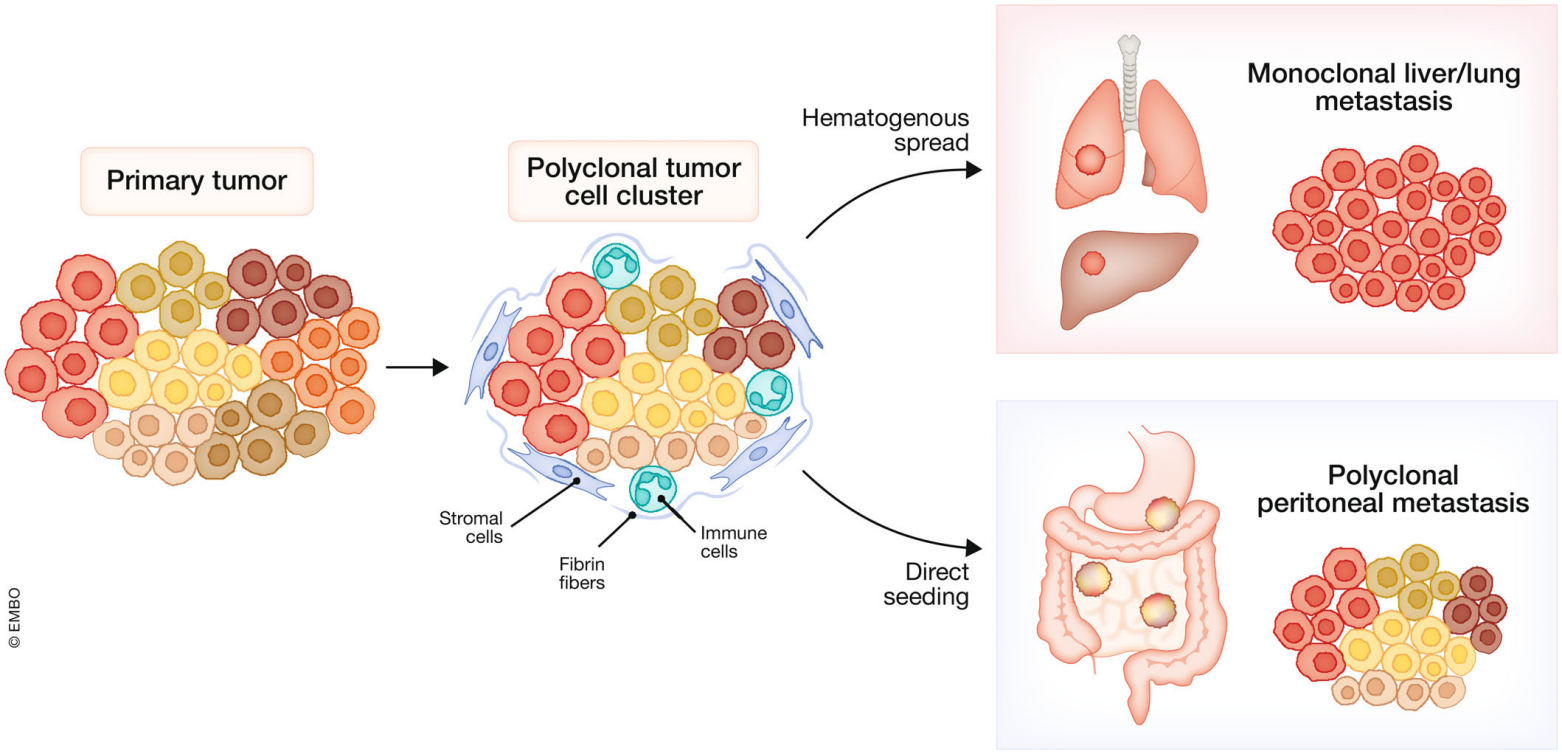
Polyclonal seeding and outgrowth of peritoneal metastases Polyclonal cancer cell clusters shed from the primary tumor into the peritoneal cavity, bloodstream or lymphatic system, where they can interact with immune and stromal cells. The clonal heterogeneity of the primary tumor is retained in peritoneal metastases, while clonal selection leads to monoclonal outgrowth of lung and liver metastases. Based on Lenos *et al* (2022) and Maddipati and Stanger (2015).

A growing body of evidence suggests that tumor cells do not necessarily travel as single cells during the metastatic process, but often as clusters that can include stromal cells and immune cells such as neutrophils (Duda *et al*, 2010; Aceto *et al*, 2014; Szczerba *et al*, 2019). Multicellular GC ascites‐derived cell aggregates indeed showed an increased metastatic potential as compared to individual cells (Dong *et al*, 2019). Cluster formation is mediated by adhesion molecules such as integrin α_v_β_3_, which also confers resistance to anoikis (Dolinschek *et al*, 2021). Tumor cell clusters with an inverted apico‐basolateral polarity (apical side out) can be found in peritoneal effusions of patients with PMD and are associated with poor prognosis (Zajac *et al*, 2018; Canet‐Jourdan *et al*, 2022). These inverted structures could already be detected in the primary tumor, suggesting collective invasion as the mechanism of dissemination. Additionally, polyclonal cancer cell clusters mediated by fibrin fibers were detected in multicolor lineage tracing models of PM in mice, in contrast to monoclonal outgrowth of liver and lung metastases (Maddipati & Stanger, 2015; Miyazaki *et al*, 2023). Similarly in CRC, PM patient samples were found to retain the clonal heterogeneity of the matching primary tumor, as opposed to liver metastases that showed evidence of monoclonal seeding (Lenos *et al*, 2022). This supports a model where polyclonal clusters shed from the primary tumor and are able to survive in the peritoneal cavity to grow out in the peritoneum (Fig 3).

### Attachment and outgrowth

The capacity of cells to adhere to the peritoneal niche is key to survival and outgrowth in the peritoneal cavity. The attachment of tumor cells to the mesothelium requires the presence of adhesion‐regulating proteins. Glycosaminoglycans such as hyaluronic acid on mesothelial cells play a vital role in the adhesion process, by binding to CD44 on the tumor cells (Harada *et al*, 2001). Furthermore, integrins on tumor cells bind to mesothelial adhesion molecules (ICAM1, VCAM1), and extracellular matrix factors in the peritoneum including fibronectin, laminin, and collagens (Sluiter *et al*, 2016). Especially β1 integrin subunits are important in peritoneal attachment, and anti‐β1 integrin antibodies successfully prevent PM formation *in vivo* (Oosterling *et al*, 2008). Collagens in the omental premetastatic niche were shown to interact with integrin α2, which is highly expressed in PM forming cancer cells (Huang *et al*, 2020). In addition to CD44 and integrins, a number of adhesion‐regulating proteins have been identified to be involved in PM formation, including L1CAM in ovarian cancer (Arlt *et al*, 2006) and Moesin in CRC (Lenos *et al*, 2022). This provides opportunities to develop therapies directed at the adhesion process. Once attached, tumor growth is supported by the activation of angiogenesis. Furthermore, mesothelial cells contribute to the creation of a tumor‐promoting niche by transitioning to cancer‐associated fibroblasts via mesothelial‐to‐mesenchymal transition (Sandoval *et al*, 2013) and secreting factors including PAI‐1, which activates the oncogenic transcription factor STAT3 on cancer cells (Hendrikson *et al*, 2022). Thus, metastatic cancer cells modify the microenvironment to allow their outgrowth.

### Peritoneal immune system

**Figure 4:**
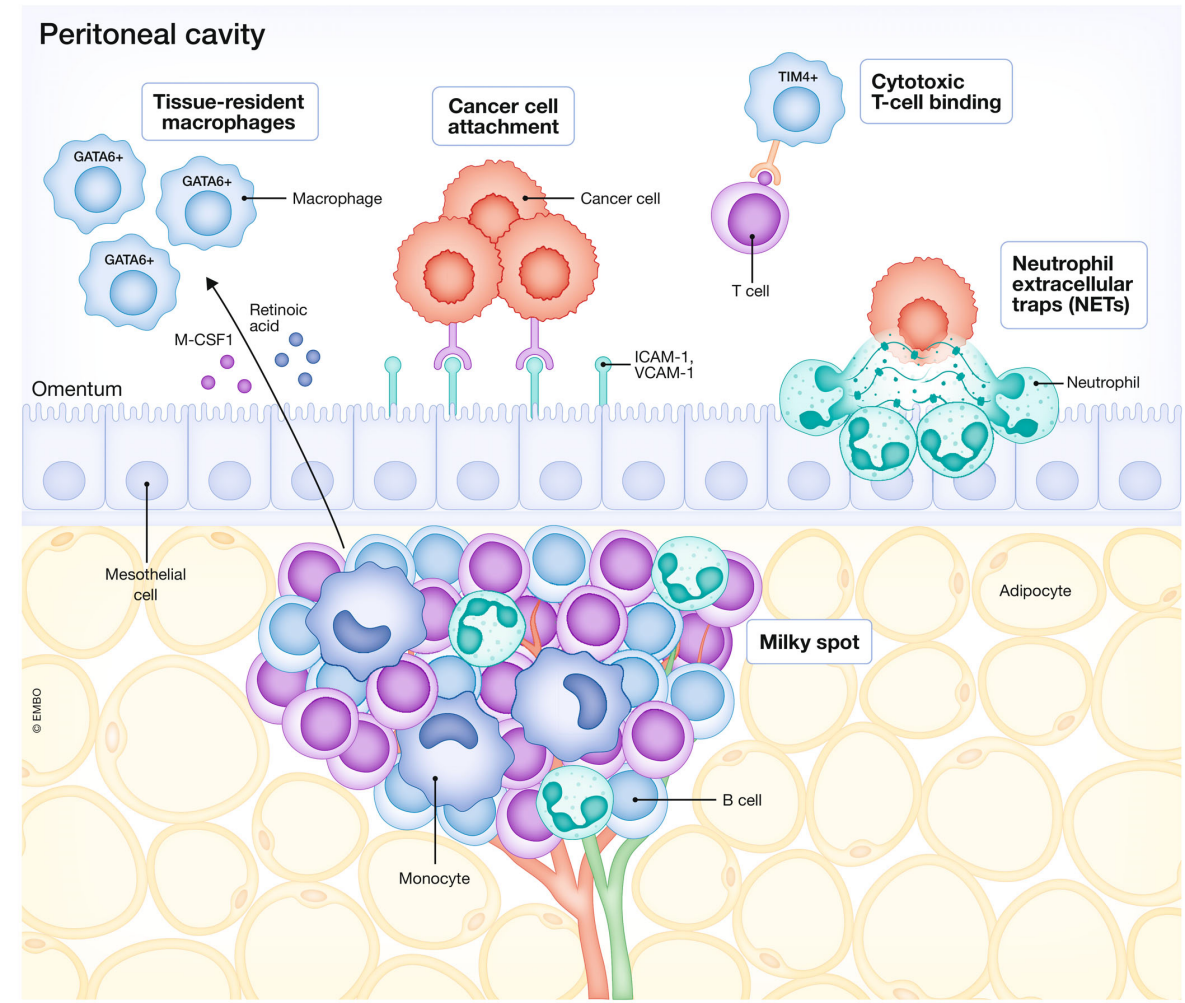
Milky spots are a key component of the peritoneal immune system Milky spots in the omentum contain various immune cells and dense capillary networks. Increased expression of cell adhesion molecules ICAM‐1 and VCAM‐1 leads to enhanced cancer cell binding. Monocytes differentiate into tissue‐resident macrophages, that can contribute to an immunosuppressive niche by binding to cytotoxic T cells. Neutrophil extracellular traps (NETs) contribute to the formation of omental metastases by creating a pre‐metastatic niche. Created with BioRender.com.

The peritoneal cavity features a unique immune cell composition, which needs to be favorable enough for metastatic cells to thrive. It is characterized by an abundance of tissue‐resident macrophages that differentiate under the influence of M‐CSF1 and retinoic acid in omental milky spots (Ratajczak *et al*, 1987; Okabe & Medzhitov, 2014) and contribute to the formation of an immunosuppressive niche during PM formation (Fig 4). As such, TIM‐4^+^ cavity‐resident macrophages directly prevent cytotoxic T cells from killing the tumor cells and thus promote metastatic spread (Etzerodt *et al*, 2020; Chow *et al*, 2021). Furthermore, GATA6^+^ peritoneal macrophages promote the growth of CRC liver metastases that breached the visceral mesothelium via immunosuppression by PD‐L1 upregulation (Hossain *et al*, 2022). Also, an increased proportion of tumor‐promoting M2‐macrophages was found in peritoneal fluid of GC patients with PMD (Yamaguchi *et al*, 2016). These findings could explain why PMD with ascites is a negative predictive biomarker for response to immune checkpoint inhibition (ICI) (Fuca *et al*, 2022).

Extensive characterization of the adaptive immune response during PM formation and outgrowth has yet to be performed. In GC PM, a T cell exclusive and T cell exhausted subgroup was identified (Wang *et al*, 2020). Immune checkpoint TIM‐3 was highly expressed in the T cell exhausted subgroup, which can be targeted by anti‐TIM‐3 antibodies that are currently being tested in Phase I studies (Wolf *et al*, 2020). In CRC, a decrease in CD4^+^ T helper and CD8^+^ cytotoxic T cells, but a marked increase in NK cells was seen in PM versus primary tumors (Seebauer *et al*, 2016). These data indicate that the peritoneal cavity has a distinct immune system, both innate and adaptive, which affects the development of intraperitoneal tumors and the response to ICI. Resensitization to ICI might be achieved by combination therapies targeting macrophages, immune checkpoints such as TIM‐3 or STING signaling (Lee *et al*, 2021). Moreover, the peritoneal immune system can be activated by intraperitoneal administration of oncolytic viruses, which is currently being tested in early phase clinical studies (Zhang *et al*, 2022a).

## Molecular features of PM

### Histology

Specific histological features associate with PM. For example, in CRC, PM occur more often in primary tumors classified as mucinous carcinoma (MC) or signet ring cell carcinoma (SRC), compared with classical adenocarcinoma histology (Hugen *et al*, 2014; Zhang *et al*, 2022b). Similarly, in GC, PM are twice as common in SRC compared with adenocarcinomas (Riihimäki *et al*, 2016). Additionally, patients with a diffuse type tumor (poorly cohesive, SRC phenotype) according to the Lauren classification, have a higher risk of presenting with PM than patients with an intestinal type tumor (well or moderately differentiated, tubular/papillary architecture) (Koemans *et al*, 2020; Verstegen *et al*, 2020). Defining the histological subtype is relevant for treatment decision making, as SRC is associated with lower survival rates and poor response to CRS‐HIPEC treatment (Razenberg *et al*, 2015; Solomon *et al*, 2019).

### Genomics

In recent years, molecular analyses have increasingly complemented histopathological assessments of tumors. In a study comparing 617 primary CRC and 348 unmatched CRC PM patients, *KRAS* and *BRAF* mutation rates were found to be similar (Stein *et al*, 2020). Fewer *APC* mutations were found (76% primary vs. 48% PM), which was confirmed in two other CRC PM cohorts (Baratti *et al*, 2021; Lenos *et al*, 2022). Mutations of other known drivers of CRC such as *TP53*, *SMAD4*, and *PIK3CA* are occasionally reported for CRC PM in literature (Lund‐Andersen *et al*, 2021), but low patient numbers make interpretation of these results difficult. In patients undergoing CRS‐HIPEC, *BRAF* mutations are mostly reported to be a negative prognostic factor (Schneider *et al*, 2018; Graf *et al*, 2020; Baratti *et al*, 2021; Di Giorgio *et al*, 2021; Flood *et al*, 2022; Tonello *et al*, 2022), although other studies report no survival difference between patients with *BRAF* mutant and *BRAF* wild‐type tumors (Massalou *et al*, 2017; Larsen *et al*, 2022). *RAS* mutations were also associated with poor prognosis in some studies (Schneider *et al*, 2018; Arjona‐Sanchez *et al*, 2019; Diez‐Alonso *et al*, 2021), while no difference was reported by others (Graf *et al*, 2020; Baratti *et al*, 2021; Larsen *et al*, 2022; Tonello *et al*, 2022). Additionally, *RAS* mutation status was a predictive marker for peritoneal recurrence, but not for systemic recurrence after CRS‐HIPEC treatment (Breuer *et al*, 2021). The discrepant results on the prognostic role of *RAS/BRAF* status in CRC PM are likely due to patient selection or low patient numbers. Also, the proportion of patients with a microsatellite instable (MSI) tumor within the *BRAF* mutant group can influence outcomes (Larsen *et al*, 2022). Lastly, the method of mutation analysis and which variants were analyzed could influence the results, for example *KRAS* G12V was associated with poorer prognosis than other *KRAS* mutations (Flood *et al*, 2022). Important to note is that the studies reporting mutation status almost all included CRS‐HIPEC treated patients, which is only a small fraction of all patients with CRC PM (Bakkers *et al*, 2021).

In GC, a higher frequency of *CDH1* (E‐cadherin) mutations was found in PM samples compared with primary tumors (26 vs. 9%) (Wang *et al*, 2020). This is in line with the association between the diffuse subtype and the formation of PM, since this subtype is strongly associated with *RHOA* and *CDH1* mutations (Cancer Genome Atlas Research Network, 2014; Kakiuchi *et al*, 2014). Furthermore, germline mutations in *CDH1* lead to a 67–83% lifetime risk of developing diffuse type GC, and an increased risk of developing lobular subtype breast cancer, which is strongly associated with PM formation as well (Pharoah *et al*, 2001; Inoue *et al*, 2017). *TP53* was the most common mutated gene in GC PM (41%); however, a similar frequency is seen in primary GC (Wang *et al*, 2020). A different study profiling malignant ascites samples confirmed the high frequency of *TP53* pathway alterations (Tanaka *et al*, 2021). *CDH1*, *TP53*, *ARID1A*, *RHOA*, *KRAS*, and *PIGR* were identified as significant driver genes. Furthermore, *Receptor Tyrosine Kinase* (*RTK*)/*RAS*/*MAP kinase* (*MAPK*) signaling pathway genes were amplified in a large part of the PM cohort, but not in previously profiled primary GC cohorts.

Although there are organ‐specific genomic features that contribute to the formation and progression of PM, commonalities can be found. As such, *RAS* mutations in CRC and amplifications in GC are present in a significant part of tumors forming PMs. To the best of our knowledge, there are no reports on mutation analysis specifically in PDAC PM, but more than 90% of primary tumors harbor a *KRAS* mutation (Witkiewicz *et al*, 2015), making the *RAS* signaling cascade an important potential therapeutic target for PM. In conclusion, mutation analysis in PM has provided important insights, however, to fully grasp the tumor biology of PM more advanced molecular characterization is needed.

### Transcriptomic subtypes

Major efforts have been made to define molecular subtypes based on gene expression. Although some subtypes are organ‐specific (e.g., EBV subtype in GC), striking similarities between subtypes of different gastrointestinal cancers can be found (Bijlsma *et al*, 2017). A poor prognosis mesenchymal subtype is present in primary CRC, GC, and PDAC tumors (Cristescu *et al*, 2015; Guinney *et al*, 2015; Dijk *et al*, 2020), which is strongly associated with PM. This subtype is characterized by downregulation of *CDH1*, upregulation of genes associated with epithelial‐mesenchymal transition (EMT), and increased TGF‐β signaling.

In GC, PM occur more frequently in the EMT subtype. In two independent datasets, the first site of recurrence of patients with a primary tumor of the EMT subtype was in 64% of the cases the peritoneum, compared to 23% in other subtypes (Cristescu *et al*, 2015). In CRC, the CMS4 subtype is associated with upregulation of mesenchymal gene expression (Guinney *et al*, 2015). In four independent cohorts, CRC PM samples classified almost exclusively as CMS4 (71–100%) (Hallam *et al*, 2020; Narasimhan *et al*, 2020; Laoukili *et al*, 2022; Lenos *et al*, 2022). In addition, the majority of matched primary tumors was also classified as CMS4, and a higher incidence of PM was found in a cohort of primary CMS4 tumors (Hallam *et al*, 2020; Laoukili *et al*, 2022; Lenos *et al*, 2022). This suggests that the subtype of the primary tumor defines the ability to metastasize to the peritoneum. Discrepant results on CMS distribution in PM samples have also been reported, which are most likely explained by the CMS classification method (Barriuso *et al*, 2021). In PDAC, no relation between molecular subtype and dissemination pattern is described yet, although *HAPLN1* was found to be a driver for PM in PDAC, and associated with the poor prognosis basal subtype (preprint: Wiedmann *et al*, 2022). In contrast to PM, liver metastases are mostly classified as the epithelial CMS2 subtype in CRC (Pitroda *et al*, 2018) and are associated with the MSS/TP53‐ and MSI subtypes in GC (Cristescu *et al*, 2015). The mesenchymal subtype enrichment thus seems to be specific for PM and may provide leads for the development of therapies targeted specifically at this subtype.

Also within PM samples, molecular subtypes have been defined. By analyzing genomic and transcriptomic data of 44 GC PM samples, a mesenchymal‐like and epithelial‐like subtype was identified (Wang *et al*, 2020). The mesenchymal‐like subtype showed TGF‐β pathway activation and T cell exhaustion, and responded poorly to chemotherapy (response rate 31 vs. 71% in the epithelial‐like subtype). Similarly, an EMT and non‐EMT subtype was found in a study profiling 59 cell lines derived from malignant GC PM ascites samples (Tanaka *et al*, 2021). The EMT subtype was associated with a poorer prognosis and TGF‐β and Hippo pathway activation. This last pathway was successfully targeted in an *in vivo* GC PM model.

Within CMS4 subtype CRC PM samples, significant heterogeneity is observed that can be categorized in three subgroups: CMS4.PM‐A (*RAS* mutated), CMS4.PM‐B (mucinous phenotype), and CMS4.PM‐C (high immune infiltration) (Lenos *et al*, 2022). As in GC, this subdivision might help identify personalized therapies based on tumor characteristics.

**Figure 5:**
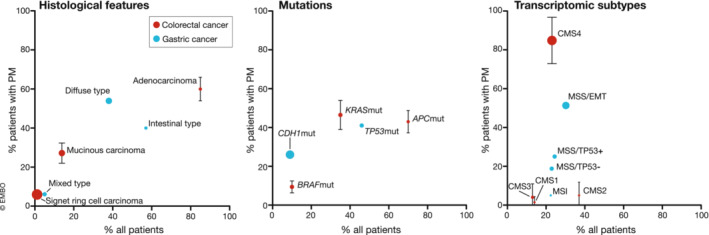
Molecular features enriched in peritoneal metastases (PM) Frequency of histological (Kerscher *et al*, 2013; Hugen *et al*, 2014; Koemans *et al*, 2020), genomic (Schneider *et al*, 2018; Graf *et al*, 2020; Stein *et al*, 2020; Wang *et al*, 2020; Baratti *et al*, 2021; Breuer *et al*, 2021; Di Giorgio *et al*, 2021; Diez‐Alonso *et al*, 2021; Flood *et al*, 2022; Lenos *et al*, 2022; Tonello *et al*, 2022) and transcriptomic (Cristescu *et al*, 2015; Narasimhan *et al*, 2020; Laoukili *et al*, 2022; Lenos *et al*, 2022) features in patients with PM from gastric cancer or colorectal cancer versus reference cohorts (Cancer Genome Atlas Research Network, 2014; Guinney *et al*, 2015). Size of the symbols reflects relative enrichment in PM.

The main histological, genomic and transcriptomic features that are discussed in this review are summarized in Fig 5. The strong association between mesenchymal subtypes and PMD calls for treatment strategies targeted at this subtype. Drug screens in cell lines that are classified as mesenchymal have been performed to identify effective compounds. In CRC, CMS4 cell lines showed a strong sensitivity to HSP90 inhibitors (Sveen *et al*, 2018). In GC, the NAMPT inhibitor FK866 was found to selectively kill GC cells with an EMT gene expression signature, which could be explained by loss of *NAPRT* gene expression in the EMT subtype (Lee *et al*, 2018). Another group identified PI3K‐inhibitors to be specifically effective against GC cell lines classified as mesenchymal (Lei *et al*, 2013). In EMT subtype cell lines from ascites‐disseminated GC, activation of the YAP‐TAZ‐TEAD pathway was seen, and administration of the TEAD inhibitor K‐975 led to *in vivo* tumor growth inhibition (Tanaka *et al*, 2021). In conclusion, although the mesenchymal subtype is characterized by drug resistance against classical chemotherapeutics, the activation of an EMT‐like program is associated with potentially targetable genes or pathways that can be exploited to develop new PMD treatment regimens.

## Discussion and future directions

There are no curative treatments available for the majority of PM patients. For patients with limited tumor burden, CRS‐HIPEC is a treatment option, although the added benefit of HIPEC to CRS is debated. In CRC, the PRODIGE 7 trial did not find a survival benefit in patients treated with CRS‐HIPEC with oxaliplatin compared to CRS alone (Quénet *et al*, 2021). This lack of effect could be explained by resistance of the mesenchymal‐subtype cancer cells to the used chemotherapeutics (Song *et al*, 2016). *In vitro* studies indeed showed cell death resistance in CMS4 cell lines (Linnekamp *et al*, 2018), and CMS4 PM‐derived organoids (Laoukili *et al*, 2022). Additionally, a meta‐analysis showed that in metastatic CRC CMS4 tumors, an oxaliplatin‐based regimen was inferior to an irinotecan‐based regimen (Ten Hoorn *et al*, 2022). The results from the Phase I INTERACT trial, where irinotecan is administered repeatedly via an intraperitoneal port in patients with CRC PM, are therefore awaited with great interest (De Boer *et al*, 2019).

For future drug development efforts, an approach where PMD‐specific features are considered will likely lead to more tailored therapies. This can involve the peritoneal niche (mesothelial attachment and the peritoneal immune system) and cancer cell‐intrinsic features (RAS signaling, mesenchymal subtype). Patients with PMD are currently underrepresented in clinical trials (Tseng *et al*, 2017). In our opinion, the PMD subgroup is ideally suited for experimental studies, since therapies can be applied locally, longitudinal sampling is possible and there is potential for improvement in terms of survival outcomes. We envision that better treatment outcomes for PMD patients will result from considering PMD as a distinct disease entity, rather than just as a secondary manifestation of their initial tumor.

## Author contributions

**Sanne Bootsma:** Conceptualization; writing – original draft; writing – review and editing. **Maarten F Bijlsma:** Conceptualization; writing – original draft; writing – review and editing. **Louis Vermeulen:** Conceptualization; writing – original draft; writing – review and editing.

## Disclosure and competing interests statement

LV received consultancy fees from Bayer, MSD, Genentech, Servier and Pierre Fabre, but these had no relation to the content of this publication. MFB has received research funding from Celgene, Lead Pharma, and Frame Therapeutics and has received consultancy fees from Servier. SB declares no conflict of interest.

**Pending issues**

- Gain more insight into the composition of the PM niche, including the immune system.
- Enhance molecular characterization of PMD, including patients with extensive disease.
- Develop improved treatment strategies based on PMD biology.
